# Translation and Cross-Cultural Adaptation of the Instrument for the Diagnosis of the Complexity of Palliative Care Needs

**DOI:** 10.1089/pmr.2024.0065

**Published:** 2025-04-22

**Authors:** Filippo Canzani, Sara Alquati, Francesca Bordin, Christian Barillaro, Marta De Angelis, Grazia Di Silvestre, Sabrina Dispenza, Gino Gobber, Caterina Magnani, Pietro Manno, Fiammetta Cosci, Silvia Tanzi

**Affiliations:** ^1^ UFC Cure Palliative, AUSL Toscana Centro, Firenze, Italy.; ^2^ Unità Cure Palliative, Azienda USL-IRCCS, Reggio Emilia, Italy.; ^3^ Unità di Cure Palliative, Hospice Dipartimento di Oncologia e Cure Palliative INI Divisione Grottaferrata, Roma, Italy.; ^4^ Centro di Continuità Assistenziale e Fragilità, Fondazione Policlinico Gemelli—IRCCS, Roma, Italy.; ^5^ UCP Cure Palliative Hospice La Torre sul Colle Spoleto, UslUmbria2, Spoleto, Italy.; ^6^ UOS Coordinamento della Rete di Cure Palliative e UVP distrettuali, ASP Palermo, Palermo, Italy.; ^7^ UO Cure Palliative, APSS Trento, Trento, Italy.; ^8^ Cure Palliative, Area governo della Rete, ASL Roma 1, Roma, Italy.; ^9^ UOC Cure Palliative, ULSS 8 Berica, Vicenza, Italy.; ^10^ Dipartimento di scienza della salute, Università di Firenze, Firenze, Italy.

**Keywords:** complexity, IDC-Pal, palliative care, patient needs, validation

## Abstract

**Background:**

In recent years, the palliative care (PC) paradigm is evolving from a prognosis-based approach to one centered on complexity, also in response to the aging population and the increase in chronic diseases. It is therefore necessary to strengthen PC networks with effective management of the specialist resources available. The use of tools such as the spanish Diagnostic Instrument for Complexity in Palliative Care (IDC-Pal) can help evaluate the complexity of PC needs, thus guiding the clinical care response. The aims of this study were the translation and the cultural adaptation of the IDC-Pal tool to the Italian language.

**Methods:**

The methodology proposed by Beaton et al. and Sousa et al. was used for the translation and cultural adaptation of the IDC-Pal tool. Phase 1: a forward–backward translation with linguistic and cultural adaptation of the tool by two native Spanish translators and two native Italian translators, including two PC professionals and two nonprofessionals, was performed. Phase 2: the translation was evaluated by a panel of 12 Italian PC experts, who assessed the comprehensibility of the translated instrument, and proposed changes to the text, which was found to be incomprehensible to at least 20% of them. Phase 3: this version of the tool was proposed to a sample of the Italian target population (93 professionals including general practitioners, nurses, and hospital doctors at 9 Italian PC networks tested it on 168 patients in home and hospital settings), to evaluate its comprehensibility and usability. At the end of the experimental phase, a semi-structured interview was organized with the main researcher of each network, with the aim of receiving information about the comprehensibility of the tool. Finally, a definitive version was developed.

**Results:**

The translation and adaptation were achieved without major problems.

**Conclusions:**

A conceptually, culturally, and linguistically equivalent italian version of the original IDC-Pal was obtained.

## Introduction

In high-income countries, 69%–82% of the population is estimated to have palliative care (PC) needs before death.^1,2^ Providing adequate PCs is a major challenge for health systems, considering also the progressive aging of the population and the increase in mortality from chronic diseases. Current resources do not allow all patients with PC needs to be adequately assisted by PC teams.^2–4^ The multidimensional needs of patients and their families could be addressed through a model of simultaneous and shared care in which the different levels and types of care are integrated and coordinated.^5,6^ In such a model, when low complex needs are present, base-level PCs would be provided by primary care services with basic palliative skills (e.g., general practitioners [GPs], medical doctors in different disciplines, community nurses), whereas more complex needs would be satisfied by multidisciplinary PC teams. However, in clinical practice, it might be difficult to identify patients who would benefit from the provision of advanced care resources; and the decision is often made subjectively, according to the skills of the specific physician taking care of the patient.^7^ Therefore, it is essential to identify a method for assessing the complexity of PC needs, which allows for an adequate distribution of health care resources, proportional to the complexity of needs, which also allows personnel with basic PC skills to identify the complexity of such needs.^4,8,9^

According to many authors, complexity is a multifactorial concept that includes elements dependent on (a) the patient, that is, clinical, social, economic, psychological, and spiritual factors; (b) the family, in terms of ability to adapt to the patient’s status; and (c) difficulties in the care, that is, the care setting, the social environment, and the political and economic situations.^8,10–12^

In recent years, the literature has raised the problem of complexity^8,10,11,13,14^ and suggested the *Instrumento Diagnóstico de la Complejidad en Cuidados Paliativos* (IDC-Pal [Diagnostic Instrument of Complexity in Palliative Care]) as a valid tool for complexity in PC assessment, to be used following the identification process of patients with palliative needs.^9,15–19^

IDC-Pal, which was created in Spain, is a diagnostic tool developed as part of the Andalusian Palliative Care Plan and is in use in several care settings.^19–22^ IDC-Pal allows to identify the elements of complexity derived by the multidimensional evaluation of the patient–family unit. Thirty-five items are presented, and they pertain to three dimensions: the patient, the family, and the health care organization. One part is devoted to a glossary that helps clarify the meaning of the items. Each item is classified as “complex” or “highly complex” (see Table 1 for the list of items and the associated level of complexity). The resulting overall level of complexity can be “noncomplex” (i.e., absence of complex or highly complex items), “complex” (i.e., presence of one or more complex items, but no highly complex items), and “highly complex” (i.e., presence of at least one highly complex item). In the case of a noncomplex status, usual care may be maintained (evaluating the start of an advance care planning process and a progressive remodulation of interventions), whereas in the case of a complex status, the PC team may become necessary depending on the level of complexity. In a highly complex status, the intervention of the PC team could be mandatory. The tool is administered by health care professionals, and direct patient involvement is not required (it is not a patient-reported outcome measure [PROM]). Spanish authors recommend knowing the case for at least three days. In fact, it is not a useful tool in acute settings. IDC-Pal was developed primarily for use by professionals who are not specialists in PC.

**Table 1.** Schedule of Interviews Conducted with the Principal Investigator of Each Palliative Care Center

Were the items understandable?
Indicate which items were most difficult to understand and why.
For the less understandable items, did reading the glossary clarify the definition?
Was the tool useful in clinical care management and why?

Considering that no instruments assessing complex PC needs are available in Italian, the aim of the present study is to provide a preliminary translation and cultural adaptation of the IDC-Pal tool in Italian. We have chosen to validate this tool, among the few available in the literature for this purpose, as it is aimed at patients suffering from all chronic diseases, not only patients with cancer (such as the Predictive Model of Complexity in Palliative Care [PALCOM] tool).^14^

## Methods

**FIG. 1.**
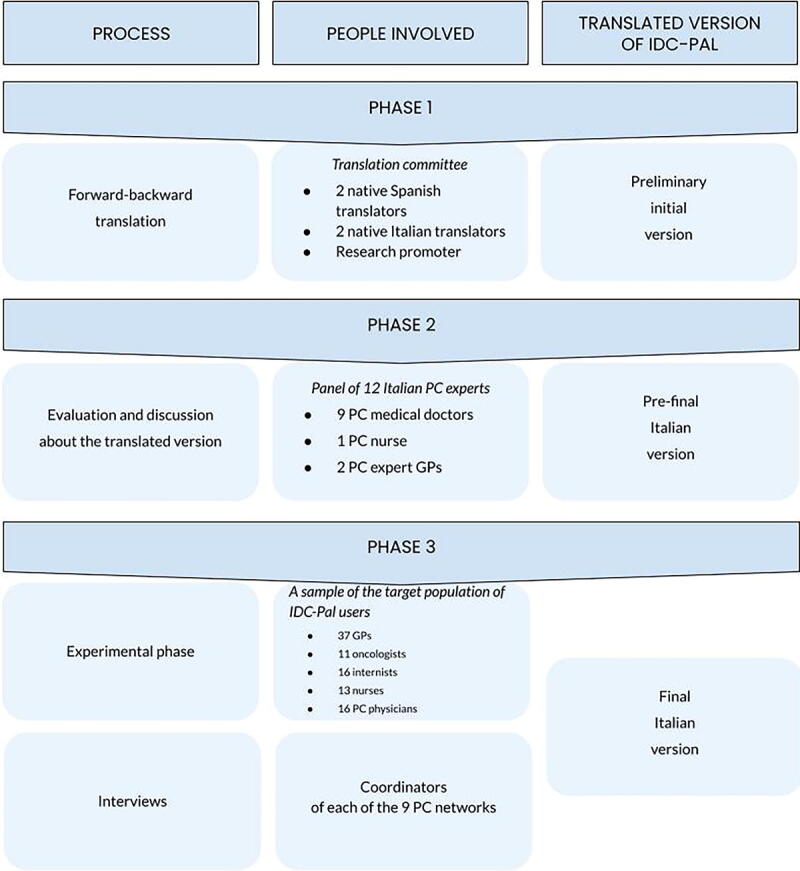
Workflow diagram for the validation process.

The methodology proposed by Beaton et al. and Sousa et al. was used for the translation and cultural adaptation of the tool (adaptations were made based on issues related to context, wording, and the structure of the items).^23,24^ The study was structured in three phases (Fig. 1).

### PHASE 1: Translation

The original Spanish instrument was translated into Italian by two independent Italian mother-tongue translators. They were proficient in both languages (CEFR - C level): One was a PC health care professional, and the second was a Spanish language teacher. Each translator filled in a report on critical issues raised during the translation. Two Italian versions of the tool were produced with slightly different nuances of Italian culture. The two versions were compared with each other and with the original one, to reach a preliminary initial translation. Ambiguities and linguistic discrepancies were discussed and solved by a translation committee, which included the two translators and the medical principal investigator of the study.

The translated version was back-translated into Spanish by two independent native Spanish translators, with the same qualifications described above. The back-translation into Spanish was blinded to the original version. This phase led to the production of two versions in Spanish, which made it possible to clarify words and sentences used in the Italian version. The two Spanish versions were then compared with each other and with the original instrument by a second translation committee, made of the four translators and the principal investigator. Ambiguities and discrepancies on meaning, colloquialisms, and idioms were discussed and solved through consensus among the committee members. Thereafter, the preliminary initial translation text was finalized.

### PHASE 2: Panel of experts

The final preliminary initial translation and the forms of the translations were subsequently evaluated by a panel of 12 Italian PC experts (i.e., 9 PC medical doctors, 1 PC nurse, and 2 PC expert primary care doctors). Each expert was asked to assess, through an online form created ad hoc, the Italian version of the tool using a dichotomous scale (“understandable”/“not understandable”). For the items rated as “not understandable,” the experts were asked to specify the reason of not understanding and provide suggestions on how to modify them. Items that were considered not understandable to more than 20% of the sample had to be rephrased. The minimum agreement required among the experts had to be 80%. A high threshold of intelligibility was chosen since Spanish and Italian are languages with strong lexical similarity (about 82%) and mutual intelligibility, as well as the two cultures of origin.^25^ This step was used to support the conceptual, semantic, and content equivalence of the translated tool and improve the structure of the sentences used in the Italian text to be easily understood by the target population. Each expert was also asked to answer, for each item, the question: “Does the corresponding glossary entry help you understand the meaning of the sentence? If NOT, why?” Finally, two online meetings among the members of the expert panel and the principal investigator were organized to discuss the items understandable by less than 80% of the panel and modify them until a unanimous agreement was reached. This led to the production of a prefinal Italian version.

### PHASE 3: Experimental phase and interviews

The prefinal Italian version was proposed to health care professionals, offering PCs to patients to assess its comprehensibility. The health care professionals were reached via nine Italian PC networks from the north to the south of Italy (Florence, Palermo, Reggio Emilia, Rome with three centers, Spoleto, Trento, Vicenza). A sample of 12 health care professionals was identified at each PC network (4 GPs, 2 oncologists, 2 internal medical doctors, 2 community nurses, and 2 PC medical doctors), by random extraction of the network members.

Each health care professional was asked to evaluate two patients progressively identified with PC needs using the prefinal Italian version of the tool. The PC specialists, at each center, reviewed all the patients evaluated by the other professionals; because in parallel with the validation study, an analysis was also conducted on the concordance between the PC valuer and the nonspecialist, using IDC-Pal on the same patient (whose data will be part of a future publication). The operators then had to report their opinions on the comprehensibility of the text and the use of the tool in their activity to their network coordinator, according to the scheme in Table 1.

The NECPAL tool^26^ was proposed to identify patients with PC needs. This is a Spanish tool whose objective is the early identification of patients with palliative needs, through an initial prognostic assessment at one year, in addition to the evaluation of some general and pathology-specific worsening criteria. This tool was chosen as it is mainly used in Italy for this purpose.

Finally, online interviews conducted by the principal researcher with the coordinators of each of the nine PC networks were organized. The purpose of the interviews was to verify the comprehensibility of the elements and the glossary of the tool, as well as the opinion on the practical use of the tool, according to the scheme proposed to health care professionals (Table 1).

**FIG. 2.**
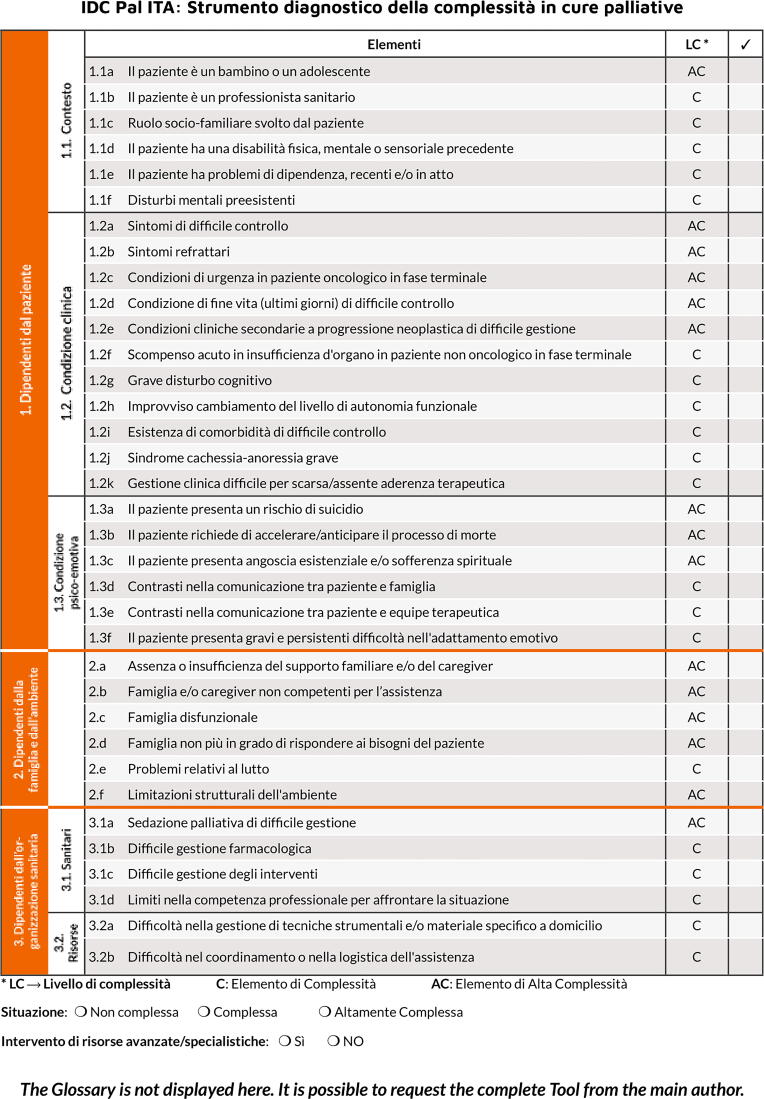
The Instrument for the Diagnosis of the Complexity of Palliative Care Needs tool translated into Italian.

This last phase led to the development of the final Italian version of the IDC-Pal tool (Fig. 2).

### Ethics

The study protocol was approved by the Local Ethical Committees of the research centers. A written consent to the study participation was obtained from all participants. The authors assert that all procedures contributing to this work comply with the ethical standards of the relevant national and institutional committees on human experimentation and with the Helsinki Declaration of 1975, as revised in 2013.

## Results

### PHASES 1 and 2

Table 2 shows data that emerged from the translation and evaluation by the panel of experts.

**Table 2.** Comprehensibility (in Percentage) Expressed by the Panel of Experts (*n* = 12) on the Items and Glossary of the Instrument for the Diagnosis of the Complexity of Palliative Care Needs Tool

Item	Item complexity	Item understandability	Glossary understandability
1. Patient-dependent items			
1.1. Background			
1.1a The patient is a child or adolescent	HC	91.7%	83.3%
1.1b The patient is a health care professional	C	100.0%	91.7%
1.1c Socio-family role played by the patient	C	91.7%	91.7%
1.1d The patient has a previous physical, mental, or sensory disability	C	83.3%	100.0%
1.1e The patient has addiction problems, recent, and/or ongoing	C	91.7%	91.7%
1.1f Preexisting mental disorders	C	91.7%	91.7%
1.2. Clinical condition			
1.2a Symptoms difficult to control	HC	100.0%	**75.0%**
1.2b Refractory symptoms	HC	91.7%	**75.0%**
1.2c Emergency conditions in terminal stage cancer patients	HC	100.0%	91.7%
1.2d End-of-life condition (last days) difficult to control	HC	100.0%	**50.0%**
1.2e Clinical conditions secondary to neoplastic progression that are difficult to manage	HC	91.7%	91.7%
1.2f Acute decompensation in organ failure in a noncancer patient in the terminal phase	C	83.3%	91.7%
1.2g Severe cognitive impairment	C	83.3%	91.7%
1.2h Sudden change in the level of functional autonomy	C	83.3%	83.3%
1.2i Existence of comorbidities that are difficult to control	C	83.3%	83.3%
1.2j Severe cachexia–anorexia syndrome	C	**58.3%**	100.0%
1.2k Difficult clinical management due to poor/absent therapeutic adherence	C	91.7%	**75.0%**
1.3. Psycho-emotional condition			
1.3a The patient is at risk of suicide	HC	91.7%	83.3%
1.3b The patient requests to speed up/anticipate the dying process	HC	100.0%	91.7%
1.3c The patient has existential angst and/or spiritual distress	HC	91.7%	**75.0%**
1.3d Contrasts in communication between patient and family	C	100.0%	83.3%
1.3e Contrasts in communication between patient and therapeutic team	C	91.7%	83.3%
1.3f The patient has severe and persistent difficulties in emotional adjustment	C	**75.0%**	**75.0%**
2. Items dependent on family and environment			
2a Absence or insufficiency of family and/or caregiver support	HC	100.0%	**58.3%**
2b Family and/or caregivers not competent in assistance	HC	91.7%	83.3%
2c Dysfunctional family	HC	91.7%	100.0%
2d Family no longer able to respond to the needs of the patient	HC	100.0%	83.3%
2e Bereavement issues	C	91.7%	**75.0%**
2f Structural limitations of the environment	HC	100.0%	100.0%
3. Items dependent on the health care organization			
3.1. Health care management			
3.1a Palliative sedation difficult to manage	HC	91.7%	**75.0%**
3.1b Difficult drug management	C	100.0%	91.7%
3.1c Difficult intervention management	C	83.3%	83.3%
3.1d Limits in professional competence to deal with the situation	C	83.3%	83.3%
3.2. Use of resources			
3.2a Difficulty in managing instrumental techniques and/or specific material at home	C	91.7%	83.3%
3.2b Difficulties in coordination or logistics of care	C	**75.0%**	91.7%

Values below the cutoff are in bold type.

C, item of complexity; HC, item of high complexity.

The item 1.2j “Severe constitutional syndrome” was the most critical because it refers to a condition that is not included in the Italian taxonomy. Thus, the item was changed to “Severe cachexia-anorexia syndrome” to better adapt the concept to the Italian scientific language. With regard to the glossary, the most critical elements were 1.2a “Symptoms difficult to control” and 1.2b “Refractory symptoms.” In both cases, the Italian definitions were retained not overlapping with the Spanish ones.

The glossary items reached an agreement above the threshold of 80%.

### PHASE 3: Experimental phase

The prefinal Italian version of IDC-Pal was used and evaluated by 93 health care professionals, who expressed consent to participate in the research compared with those extracted in each center: 37 (39.8%) GPs, 11 (11.8%) hospital oncologists, 16 (17.2%) internists, 13 (14.0%) nurses, and 16 (17.2%) PC physicians (Table 3).

**Table 3.** Health Care Professionals Enrolled in the Study and Patients Screened by Health care Professionals

	Enrolled health care professionals		Patients screened by professionals	
	*N*	%	*n*	%
GPs	37	39.8%	71	42.3%
Oncologists	11	11.8%	32	19.0%
Internal medical doctors	16	17.2%	38	22.6%
Nurses	13	14.0%	27	16.1%
Palliative care medical doctors^a^	16	17.2%	—	—
*Total*	*93*	*100.0%*	*168*	*100.0%*

^a^ Palliative care physicians evaluated all 168 patients.

GPs, general practitioners.

A total of 168 patients were assessed using the Italian version of the IDC-Pal, of which 83 patients (49.4%) were males, 85 (50.6%) were females; the mean age was 74.4 years (range = 28–99 years). A total of 71 (42.3%) patients were evaluated by GPs, 32 (19.0%) by oncologists, 38 (22.6%) by internists, and 27 (16.1%) by community nurses (Table 3). Palliative specialists assessed the whole sample. A total of 132 (78.6%) patients had an oncological pathology, and 36 (21.4%) had a nononcological pathology. Table 4 shows the complexity of data that emerged from the evaluation with IDC-Pal of the population sample.

**Table 4.** Results in the Use of Instrument for the Diagnosis of the Complexity of Palliative Care Needs in Terms of Complexity Assessed by Operators

	Non-PC workers		PC workers	
Result	*N*	%	*N*	%
High complexity	152	90.5%	143	85.1%
Complexity	16	9.5%	25	14.9%
*Total*	*168*	*100.0%*	*168*	*100.0%*

PC, palliative care.

### PHASE 3: Interviews

The online interviews conducted at the end of phase 3 suggested an understandability of items and glossary. The most critical items in terms of comprehensibility were “The patient presents existential anguish and/or spiritual suffering” and “The patient presents serious and persistent difficulties in emotional adaptation,” where the meaning of an item was less clear, and the glossary was able, however, to clarify it in over 95% of cases.

All the interviewees (nine, 100%) reported that IDC-PAL was a quick-to-use tool, completing it in about two minutes when they became familiar with it. Nonetheless, the negative visual impact that the tool can have at the beginning, presenting a long list of elements, was reported at three centers. They also noted that it is designed predominantly for non-PC professionals (although it also helps specialists). Seven centers reported that the tool helps communication between non-PC professionals and PC teams. According to two respondents, IDC-Pal “helps create a uniform language” among health care professionals. Eight centers indicated that the tool helps to identify the most appropriate level of PC care. Three interviewees observed that the tool allows for a “snapshot” of cases at the enrollment in PC network. Four centers suggested that the tool can help in the training of the health care personnel, especially non-PC personnel, by identifying critical areas (e.g., when the presence of symptoms that are difficult to control is detected as complexity). Table 5 summarizes some sentences about the experience of using the translated tool.

**Table 5.** Focus on Some Sentences Reported by Operators Who Tested the Translated Version of Instrument for the Diagnosis of the Complexity of Palliative Care Needs

“… quick-to-use tool, completing it in about a minute when you became familiar with it”
“It helps communication between non-palliative care professionals and PC teams”
“It helps create a uniform language”
“It’s so useful to identify the most appropriate level of PC care”
“IDC-Pal allows to have a ‘snapshot’ of cases at the enrollment in PC network”
“The tool could help in the training of the health care personnel”

## Discussion

The current study has provided a preliminary Italian version of the IDC-Pal tool. There were no deletions or additions. In phase 1, the only expression needing adaptation was “severe constitutional syndrome” as there was no direct equivalent in Italian terminology; we replaced it with “anorexia-cachexia syndrome.” In phase 2 of the study, building on the previous discussion, the most complex elements for the panel of experts, in terms of translation and understanding, concerned the psycho-emotional condition, as well as the difficulty of coordination and logistical care.

Apparently, health care professionals in PC are less familiar with the psycho-existential area, as confirmed by the intelligibility of the glossary item “the patients have existential or spiritual distress”; the item concerned the emotional adjustment in this area, which typically pertains to clinical psychologists and psychiatrists, in Italy. The introduction of a psychologist within the panel of experts could have helped, but it was mainly important to develop a translation intelligible for the target population, doctors, and nurses.

The difficulty of these items was also confirmed by the concordance data that will be published in another article; concordance on refractory symptoms, existential and social needs, as well as patient coping management was minimal among professionals compared with, for example, the identification of physical symptoms. This emphasizes the necessity to train nonspecialist professionals to recognize these items in the first place.

Other glossary items did not give any problems, we only changed “When there is insufficient control of physical and/or psycho-emotional symptoms, a long-term evolution (more than 5 days …)” into “When there is insufficient control of physical and/or psycho-emotional symptoms, a protracted agonising phase …” to make it more understandable to the panel of experts.

As for the rest, the translation did not present any problems, considering that the two languages have a strong lexical similarity.

From the interviews conducted after the experimentation, some themes emerged as shown in Table 5, confirming the instrument’s ease of use and handling.

Moreover, the IDC-Pal is recognized as a quick tool to apply (about two minutes) in an ambulatory setting, and the referrals mediated by the use of IDC-Pal were appropriate.

The tool helps to favor inter-rater reliability and to focus attention on all dimensions aligning the gaze of all professionals, experienced and nonexperienced, in PC.

Criticism from the interviews after the experiential phase revealed that the difficulties in using it were only for the first few times, while afterward, the speed of execution was appreciated.

Moreover, the terms that had been difficult to understand in phase 2 proved to be more difficult to understand in phase 3 as well, but the glossary helped to clarify them.

### Limitations

A limitation might be not having calculated the psychometric properties of the instrument; but IDC-Pal is an instrument to be used by professionals, not PROMs.

The predominance of patients with cancer (a third of the patients) could be a limitation; this reflects the Italian reality of patients recognized as most in need of PC, most of whom are still oncological.

GPs were the most represented professional class in the study group; the set of patients was evaluated by a sample of population of professionals who are possible users of IDC-Pal in clinical practice. It is expected that in Italian PC networks, the tool will be used mainly by GPs, for more correct activation of specialist resources.

Further studies to evaluate the properties of the Italian version of the IDC-Pal are warranted.

## Conclusions

The IDC-Pal Italian version is understandable and effective in clinical use, helps to identify the most suitable care path according to the multidimensional needs of PC, offers a possible standard for assessing the complexity of needs, and provides “a common language” to be adopted by professionals of different disciplines and skills. IDC-Pal requires being implemented in other countries to allow a standardized global assessment of the complexity of needs of patients in PC needs.

## Abbreviations Used

CEFR: The Common European Framework of Reference C level is for proficient users of a language, with C1 being advanced and C2 being proficiency.
IDC-Pal: Instrumento Diagnóstico de la Complejidad en Cuidados Paliativos
NECPAL: Necesidades Paliativas (Palliative Care Needs). Tool for identifying patients with advanced or terminal diseases with palliative care needs
PC: Palliative Care
PROMs: Patient-Reported Outcome Measures
